# Inappropriate trusting behaviour in dementia

**DOI:** 10.3389/fneur.2024.1433135

**Published:** 2024-09-03

**Authors:** Anthipa Chokesuwattanaskul, Dexter Penn, Claudia Albero, Jeremy C. S. Johnson, Elia Benhamou, Lucy L. Russell, Chris J. D. Hardy, Charles R. Marshall, Jonathan D. Rohrer, Jason D. Warren

**Affiliations:** ^1^Dementia Research Centre, Department of Neurodegenerative Disease, UCL Queen Square Institute of Neurology, University College London, London, United Kingdom; ^2^Division of Neurology, Department of Internal Medicine, King Chulalongkorn Memorial Hospital, Thai Red Cross Society, Bangkok, Thailand; ^3^Cognitive Clinical and Computational Neuroscience Research Unit, Faculty of Medicine, Chulalongkorn University, Bangkok, Thailand; ^4^Centre for Preventive Neurology, Wolfson Institute of Population Health, Queen Mary University of London, London, United Kingdom

**Keywords:** trust, Alzheimer’s disease, frontotemporal dementia, primary progressive aphasia, dementia

## Abstract

**Background:**

Inappropriate trusting behaviour may have significant social, financial and other consequences for people living with dementia. However, its clinical associations and predictors have not been clarified. Here we addressed this issue in canonical syndromes of frontotemporal dementia (FTD) and Alzheimer’s disease (AD).

**Methods:**

In 34 patients with AD and 73 with FTD (27 behavioural variant (bv)FTD, 22 semantic variant primary progressive aphasia (svPPA), 24 nonfluent/agrammatic variant (nfv)PPA) we recorded inappropriate trusting and other abnormal socio-emotional behaviours using a semi-structured caregiver survey. Patients were comprehensively characterised using a general cognitive assessment and the Revised Self-Monitoring Scale (RSMS; an informant index of socioemotional awareness).

**Results:**

Inappropriate trusting was more frequent in svPPA (55%) and bvFTD (44%) than nfvPPA (17%) or AD (24%). After adjusting for age, sex, education and Mini-Mental State Examination (MMSE) score, inappropriate trusting was significantly more likely in svPPA (odds ratio 3.61; 95% confidence interval 1.41–8.75) and bvFTD (3.01, 1.23–6.65) than AD. Significant predictors of inappropriate trusting comprised apathy in svPPA, disinhibition and altered pain responsiveness in bvFTD, and lower MMSE and RSMS (self-presentation) scores in AD.

**Conclusion:**

Dementia syndromes vary in prevalence and predictors of abnormal trusting behaviour, with implications for clinical counselling and safeguarding.

## Introduction

People living with dementia are at substantial risk from impaired judgment and decision making, including inappropriately placing trust in others. This is particularly pertinent to financial decisions and susceptibility to scams (1, 2). While cognitive impairment *per se* may lead to inappropriate trusting and impaired scam detection (2, 3), patients with diseases in the frontotemporal dementia (FTD) spectrum may be relatively more vulnerable due to early, prominent changes in socio-emotional behaviour and awareness (1, 4–7). However, the factors that drive abnormal trusting behaviour and how these might vary between canonical dementia syndromes have not been defined.

Here we addressed this issue in patients representing Alzheimer’s disease (AD) and the major behavioural and language-led variant syndromes of FTD (behavioural variant (bv)FTD, semantic variant primary progressive aphasia (svPPA) and nonfluent/agrammatic variant (nfv)PPA). We surveyed patients’ primary caregivers about abnormal trusting and other potentially relevant behavioural changes since illness onset, and assessed the influence of diagnosis, cognitive and behavioural features on the development of inappropriate trusting. Based on clinical experience and previous evidence, we hypothesised that inappropriate trusting behaviour would be more prevalent in FTD syndromes than AD, and would be predicted by abnormal interpretation of socio-emotional signals, impaired governing of own social behaviour, and/or altered responsiveness to aversive consequences. The last is likely to share pathophysiological mechanisms with responsiveness to pain, which is commonly altered in bvFTD and svPPA syndromes and was accordingly used here to index abnormal behavioural sensitivity to negative outcomes more generally (8, 9).

## Materials and methods

We studied 107 patients: 34 with typical amnestic AD and 73 with major FTD syndromes (27 bvFTD, 22 svPPA, 24 nfvPPA). All had compatible general neuropsychological, brain MRI and CSF findings, and mild to moderately severe disease (details in Table 1). Each patient had a primary caregiver who could provide reliable information on their premorbid and current behaviour.

**Table 1:** General demographic, neuropsychological and behavioural characteristics of patient groups.

Characteristic	AD*	bvFTD	svPPA	nfvPPA
Demographics				
No. (male:female)	34 (18:16)	27 (20:7)	22 (13:9)	24 (14:10)
Handedness (R:L)	30:4	26:1	21:1	23:1
Age (y)	70.7 (8.1)	66.5 (7.7)	66.1 (7.1)	70.9 (8.1)
Education (y)	16.0 (12.2–16.0)	14.0 (12.0–16.0)	16.0 (11.2–16.0)	13.5 (11.0–16.0)
Symptoms duration (y)	5.3 (4.2–7.6)^4^	4.7 (3.7–5.7)	5.4 (4.6–6.3)	4.3 (2.6–5.1)^1^
MMSE (/30)	18.5 (16.2–25.0)	25.0 (21.5–27.5)	23.5 (18.5–28.5)	25.5 (17.2–28.0)
WASI VIQ	97.0 (84.0–110.5)^3,4,b^	86.0 (61.0–113.0)^3,b^	65.0 (55.0–76.0)^1,2,a^	75.0 (66.0–97.0)^1,c^
WASI PIQ	83.0 (74.0–96.3)^3,b^	94.0 (83.0–109.0)^b^	111.0 (98.0–129.0)^1,a^	89.0 (77.0–106.0)^c^
Neuropsychology				
*Episodic memory*				
RMT faces (/50)	30.5 (26.0–35.3)^j^	32.0 (25.3–38.5)^a^	29.0 (27.5–36.0)^c^	34.0 (30.0–10.0)^c^
RMT words (/50)	28.5 (25.0–38.3)^j^	36.0 (27.8–44.0)^c^	31.5 (27.0–38.5)^d^	41.0 (28.0–45.0)^c^
*Executive*				
DS forward (12)	6.0 (5.0–7.0)^4,b^	6.5 (5.0–7.8)^4,a^	7.0 (6.0–7.0)^4,a^	4.0 (4.0–5.0)^1,2,3,c^
DS reverse (12)	4.0 (3.0–4.5)^c^	4.0 (3.0–5.0)^4,a^	5.0 (4.0–5.0)^4,a^	3.0 (0–4.0)^2,3,c^
WASI matrices (30)	11.0 (7.8–16.0)^3,b^	16.5 (9.0–24.0)^3,a^	25.0 (21.0–29.0)^1,2,4,a^	11.5 (6.0–22.0)^3,b^
DKEFS stroop: colour (90 s)	54.0 (45.0–61.0)^4,e^	44.0 (33.0–66.0)^4,b^	51.0 (37.0–67.0)^4,a^	90.0 (63.0–90.0)^1,2,3,g^
words (90 s)	32.0 (28.0–36.0)^4,e^	30.0 (22.0–35.0)^4,b^	28.0 (23.0–35.0)^4,a^	66.0 (52.0–90.0)^1,2,3,g^
interference (180 s)	148.0 (106.0–180.0)^3,e^	83.0 (60.0–180.0)^4,b^	92.0 (62.0–128.0)^1,4,a^	180.0 (119.8–180.0)^2,3,f^
TMT-A (s)	69.0 (58.0–127.0)^c^	56.0 (40.0–93.0)^b^	53.0 (33.0–61.0)^a^	62.0 (42.0–143.0)^g^
TMT-B (s)	300.0 (194.5–300.0)^3,c^	192.0 (100.0–300.0)^b^	115.0 (82.0–177.0)^1,a^	238.0 (149.0–300.0)^g^
Letter fluency (F)	10.0 (6.0–13.0)^c^	8.0 (2.0–14.0)^b^	7.0 (4.0–12.0)^a^	4.0 (0–8.0)^g^
Category fluency (animals)	9.0 (5.8–13.3)^b^	9.0 (5.0–17.0)^b^	5.0 (2.0–9.0)^a^	9.0 (2.0–15.0)^g^
*Language*				
BPVS (/150)	144.0 (124.8–146.3)^3,b^	141.0 (135.0–148.0)^3,b^	84 (33.0–107.0)^1,2,4,a^	139.5 (114.8–144.0)^3,b^
GNT (/30)	14.0 (4.5–20.5)^3,c^	15.0 (3.0–23.8)^3,a^	0 (0–0)^1,2,4,a^	9.0 (6.0–19.0)^3,c^
*Other skills*				
GDA (/24)	2.0 (1.0–6.5)^2,3,c^	6.0 (4.0–14.0)^1,4,d^	12.0 (5.0–16.0)^1,4,a^	3.0 (0–5.0)^2,3,c^
VOSP (/20)	16.0 (14.0–17.3)^b^	15.5 (10.0–18.0)^c^	16.0 (15.0–17.3)^b^	17.0 (14.8–18.0)^d^
*Social cognition*				
RSMS-total	34.0 (12.3)^2,f^	20.4 (11.8)^1,4,b^	23.5 (11.2)^4,d^	35.4 (18.0)^2,3,g^
RSMS-EX	15.0 (10.0–21.0)^e^	8.0 (2.0–14.0)^b^	7.5 (5.0–12.0)^d^	17.0 (4.0–23.0)^g^
RSMS-SP	18.8 (6.5)^2,e^	12.1 (5.4)^1,4,b^	14.7 (6.0)^d^	19.8(8.4)^2,g^
Obsessionality	8 (24)^2,3^	21 (78)^1,4^	13 (59)^1^	8 (33)^2^
Disinhibition	8 (24)^2,3^	23 (85)^1,4^	14 (64)^1^	7 (29)^2^
Apathy	23 (68)	22 (81)	9 (41)	12 (50)
Altered pain sense	4 (12)^2,3^	12 (44)^1^	12 (55)^1^	8 (33)
Inappropriate trust	8 (24)	12 (44)	12 (55)^1^	4 (17)

This table presents demographic, neuropsychological and behavioural characteristics of diagnostic groups, based on the complete patient cohort. Counts (standard deviation) are shown for general demographic and clinical data; mean (standard deviation) or median (interquartile range) scores are shown for neuropsychological tests (also with maximum scores in parentheses); and raw counts (percentage of group) are shown for behavioural change data. All patients fulfilled current consensus diagnostic criteria [AD: Dubois et al. (21); bvFTD: Rascovsky et al. (22); PPA: Gorno-Tempini et al. (23)]. *15 patients underwent lumbar puncture and/or brain amyloid-PET; all had biomarker profiles consistent with underlying AD pathology, based on local criteria. Significant differences between patient groups are coded as follows: ^1^significantly different from AD, ^2^significantly different from bvFTD, ^3^significantly different from svPPA, ^4^significantly different from nfvPPA (all p_FDR_ < 0.05). AD, patient group with typical Alzheimer’s disease; BPVS, British Picture Vocabulary Scale (24); bvFTD, patient group with behavioural variant frontotemporal dementia; D-KEFS, Delis Kaplan Executive System (25); DS, Digit Span; EX, sensitivity to socio-emotional expressiveness; GDA, Graded Difficulty Arithmetic test (26); GNT, Graded Naming Test (27); L, left; MMSE, Mini-Mental State Examination score (28); nfvPPA, patient group with nonfluent/agrammatic variant primary progressive aphasia; PIQ, performance IQ; R, right; RMT, Recognition Memory Test (29); RSMS, Revised Self-Monitoring Scale; s, seconds; SP, ability to modify self-presentation; svPPA, patient group with semantic variant primary progressive aphasia; Symptoms, estimated duration of symptoms since onset; TMT, trail making test; VIQ, verbal IQ; VOSP, Visual Object and Space Perception Battery – Object Decision test (30); WASI, Wechsler Abbreviated Scale of Intelligence (31). A reduced number of patients completed certain tests, as follows: ^a^n-1, ^b^n-2, ^c^n-3, ^d^n-4, ^e^n-5, ^f^n-6, ^g^n-7, ^h^n-8, ^j^n-10. The subcohort of 69 patients included in the predictor analysis (Supplementary Table S2) did not differ significantly in any cognitive or behavioural characteristic from the full cohort (see Supplementary Table S3).

In a semi-structured survey, caregivers were asked whether there had been increased instances of patients inappropriately trusting other people, such as heightened gullibility, incautiousness or acts of poor judgement, and were invited to provide examples. They were also surveyed about the presence or absence of changes in other socio-emotional behaviours (social disinhibition, obsessionality, apathy, altered pain responsiveness) that we hypothesised might be relevant to inappropriate trusting. Caregivers were asked to assess behavioural changes relative to the patients’ behaviour 10 years previously (an interval predating symptom onset for all patients). Caregivers also completed the Revised Self-Monitoring Scale (RSMS) (10) an index of social impression management and responsiveness to changes in the social environment. The scale has two subscores. The socioemotional expressiveness score (RSMS-EX) measures the ability to understand social cues of others, and the modification of self-presentation score (RSMS-SP) measures the ability to change one’s behaviour when it is not appropriate in a social situation.

Data were analysed using Python (v3.8.5) software and the logistic regression package from scikit-learn 1.2.0 with bootstrapping (10,000 iterations each) for all logistic regression analyses.

Participant groups were compared on demographic, cognitive and behavioural measures using ANOVA and Kruskal-Wallis tests for continuous variables, and chi-square tests and Fisher’s exact tests (when expected counts were small) for categorical variables. Post-hoc pair-wise comparisons were carried out when applicable, with false-discovery-rate correction. For all tests, *p* < 0.05 was accepted as the threshold for statistical significance.

Odds of inappropriate trusting behaviour in each FTD syndromic group compared to the AD group were assessed using logistic regression models, adjusting for age, sex, years of education and Mini-Mental State Examination (MMSE) score (a surrogate for disease severity). In separate univariate logistic regression models based on 69 patients with complete correlative neuropsychological and behavioural data, we assessed candidate cognitive and behavioural predictors of inappropriate trusting behaviour within different syndromic groups. These candidate predictors comprised MMSE score (overall level of cognitive function), WASI (Wechsler Abbreviated Scale of Intelligence) Matrices subtest score (nonverbal reasoning ability), RSMS-total score, RSMS-EX (socio-emotional expressiveness subscore), RSMS-SP (ability to modify self-presentation subscore) and presence (or absence) of social disinhibition, obsessionality, apathy and altered pain responsiveness.

The study was approved by the University College London institutional ethics committee and all participants gave informed consent in accordance with the Declaration of Helsinki. The data that support the findings of this study are not publicly available (in line with the terms of the original ethics approval) but available on reasonable request from the corresponding author.

## Results

Patient groups were well matched in age, sex and years of education and showed cognitive and behavioural features in keeping with their syndromic diagnoses (Table 1), including lowest RSMS and highest prevalence of social disinhibition, obsessionality and altered pain responsiveness in the bvFTD and svPPA groups.

**Figure 1:**
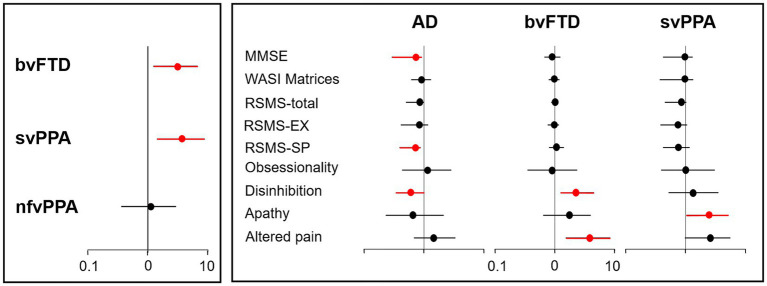
Risk factors for inappropriate trusting behaviour in dementia syndromes. The figure is a graphical representation of diagnostic, cognitive and behavioural risk factors for inappropriate trusting behaviour in patients with dementia (see Supplementary Table S2). Odds ratios (with 95% confidence intervals) are shown, plotted on a log-10 scale; significantly increased or reduced odds are depicted in red. The left panel displays the risk associated with a diagnosis of each canonical frontotemporal dementia syndrome relative to Alzheimer’s disease (adjusted for age, sex, years of education, and Mini-Mental State Examination score; see text). The right panel displays the risk associated with key cognitive and behavioural factors within each diagnostic group (excluding the nfvPPA group, as only four patients with this diagnosis showed inappropriate trusting). AD, patient group with Alzheimer’s disease; bvFTD, patient group with behavioural variant frontotemporal dementia; EX, sensitivity to socio-emotional expressiveness; MMSE nfvPPA, patient group with non-fluent/agrammatic primary progressive aphasia; MMSE, Mini-Mental State Examination score; RSMS, Revised Self-Monitoring Scale; SP, ability to modify self-presentation; svPPA, patient group with semantic variant primary progressive aphasia; WASI, Wechsler Abbreviated Scale of Intelligence.

Across all patient groups, inappropriate trusting behaviour was more prevalent in the svPPA (12/22 cases, 55%) and bvFTD (12/27, 44%) groups than the nfvPPA (4/24, 17%) or AD (8/34, 24%) groups. Risk was most significantly increased with a diagnosis of svPPA (odds ratio 3.61; 95% confidence interval 1.41–8.75) and bvFTD (3.01; 1.23–6.65) relative to AD but did not differ significantly between nfvPPA and AD (1.06; 0.36–2.96) (Figure 1). When invited to describe patients’ inappropriately trusting behaviour, a number of caregivers detailed how they had fallen victim to email and other financial “scams” (examples in Supplementary Table S1).

Significant predictors of inappropriate trusting behaviour (after adjusting for age, sex and years of education) comprised apathy (odds ratio 2.48; 95% confidence interval 1.03–5.14) and a trend for higher altered pain responsiveness (2.59; 0.97–5.58) in the svPPA group; disinhibition (2.24; 1.25–4.50) and altered pain responsiveness (3.83; 1.52–8.41) in the bvFTD group; and lower MMSE (0.71; 0.29–0.92) and RSMS-SP scores (0.71; 0.39–0.88) and absence of disinhibition (0.60; 0.34–0.99) in the AD group (Figure 1; Supplementary Table S2).

## Discussion

Our findings show that inappropriate trusting presents a significant issue in people living with both AD and FTD, and that patients with bvFTD and svPPA are at highest risk of developing this behaviour, in line with the greater prominence of socio-emotional behavioural deficits in these FTD syndromes (5, 7, 10). Predictors of abnormal trusting here varied between dementia syndromes. In keeping with previous evidence (3), overall level of cognitive impairment and inability to monitor one’s own social conduct were predictors in AD. Disinhibition and abnormal responsiveness to aversive consequences (here indexed as pain) predicted inappropriate trusting behaviour in bvFTD, while apathy was a predictor in svPPA. These profiles suggest different candidate neural mechanisms for abnormal trusting linked to particular socio-emotional behavioural abnormalities in these diseases, in line with previous work (6–8, 10, 11).

Complex behavioural changes are multi-dimensional (8). Apathy in svPPA might promote inappropriate trusting and financial vulnerability by impairing initiative, motivation and autonomous goal-setting (12, 13). However, disinhibition in bvFTD here tended to promote inappropriate trusting behaviour but in AD was relatively ‘protective’. We do not have details about how these behavioural complexes presented in our AD and FTD patients – and further, they were indexed by caregiver report. Whereas disinhibition in FTD tends to manifest as over-familiarity and lack of awareness of social cues, disinhibition and other forms of social inappropriateness in AD may be more associated with irritability, anxiety, social withdrawal, less compliance with social suggestions and wariness of novelty (14, 15). *Absence* of disinhibition in our AD group may have been a risk factor for inappropriate trusting behaviour if (in AD) *disinhibition* promotes irritability toward potential scammers, reduced social compliance and wariness of others’ suggestions. Further, the presence versus absence of disinhibition in AD and FTD may be differentially associated with other cognitive capacities (such as emotional sensitivity and decision making) that were not directly captured here.

Altered pain responsiveness might signify a more general problem with physiological anticipation, homeostasis and autonomic coding of potentially salient events, rewards and/or punishments, corroborating previous evidence in FTD syndromes (8, 9, 16, 17). Moreover, the association between pain responsiveness and decision making might be underpinned by overlapping neuroanatomical correlates involving the brain’s salience and homeostatic networks (9, 18). Blunted sensitivity to diverse kinds of negative consequences could tend to promote recurrent risk taking behaviours, reduced apprehensiveness of potentially harmful consequences and gullibility in ambiguous interpersonal exchanges. Pathological gambling can be a significant issue in FTD (19) and it is noteworthy that many caregivers here recorded substantial daily-life impacts of patients’ inappropriate trusting behaviour, notably financial exploitation (see Supplementary Table S1).

Our findings illuminate socio-emotional behavioural changes that promote vulnerability to poor financial and other decision making linked to misplaced trusting in people with major dementias. The findings add to existing evidence that links reduced cognition (1, 6), accumulation of neurodegenerative pathology (3) and cerebrovascular insults (20) to impaired decision making and financial vulnerability. This is a significant clinical issue that warrants greater awareness and understanding by clinicians, health policy makers, safeguarding authorities, financial regulators and especially, people living with dementia and their caregivers. Potential vulnerability to financial and other scams should be considered in all cognitively impaired people: indeed, even in the “lower risk” syndromic groups here (AD, nfvPPA), a substantial minority of patients had exhibited misplaced trust. However, our findings may help prioritise clinical counselling and financial safeguarding discussions where the syndromic diagnosis (bvFTD, svPPA) or behavioural profile places the patient at particularly high risk of exploitation.

This study has several limitations that should inform future work. Assessment of inappropriate trusting and other socio-emotional behaviours was based on caregiver reports, in a relatively small patient cohort. Future studies should focus on creating more objective methods for assessing these behaviours, alongside decision making capacity and financial vulnerability in people with dementia, and should assess prospectively the specific daily life impacts of inappropriate trusting and other risky behaviours on financial and social functioning, well-being and care burden. Inappropriate trust is a highly complex psychological construct: functional neuroimaging techniques such as fMRI would further understanding by elucidating underlying neural mechanisms, and clarifying how these differ (or converge) between dementia syndromes. Additionally, more detailed stratification of the neuropsychological, behavioural and neural predictors of misplaced trusting in larger and more diverse neurodegenerative disease cohorts as well as in cognitively well older people would allow development of bespoke clinical counselling and safeguarding strategies. It will also be important to establish in longitudinal studies when and how potential vulnerabilities develop over the course of the illness. As a first step, the present findings should prompt clinicians to enquire about inappropriate trusting and vulnerability to scams in all people living with dementia, with a particularly high index of suspicion in bvFTD and svPPA, and in the setting of other socio-emotional behavioural changes.

## Data Availability

The datasets presented in this article are not readily available because our ethical approvals and clinical confidentiality considerations specifically preclude public archiving of participant data. Requests to access the datasets should be directed to the corresponding author.
