# Supplementary material for: Inappropriate trusting behaviour in dementia
Source: Front Neurol. 2024 Sep 3;15:1433135. doi: 10.3389/fneur.2024.1433135 (PMC11414237; doi:10.3389/fneur.2024.1433135)
Supplement: Supplementary file 1 [file Data_Sheet_1.PDF]

Supplementary Material. Inappropriate trusting behaviour in dementia, by A Chokesuwattanaskul et al.

Table S1. Representative caregiver descriptions of inappropriate trusting behaviour

Diagnosis	Case	Caregiver comments
bvFTD	1	Taken advantage of financially
	2	Responds to mail scams, sends cheques and gives money away
	3	Taken advantage of by customers; accepted artwork as payment instead of money, doing work for free
	4	Victim of phone scams
	5	Duped - gave money for a plane ticket in response to a 'sob story'; conned out of money with a story about an unplanned pregnancy
	6	Uncharacteristically gave away lots of money to charity
	7	Vulnerable to phone scams
	8	Gave lots of money to Big Issue sellers
svPPA	1	Victim of online scams
	2	Taken in by a postal competition or email scam
	3	Victim of email scams
	4	Taken in by online scams on beauty products
	5	Victim of scam emails
	6	Misjudges scam emails/phone calls; very forgiving toward cold callers
	7	Gives money away
	8	Talked into spending a lot on cosmetics; gives money to the homeless all the time
	9	Caught by email scam; now directs all calls to spouse as never sure if genuine
	10	Vulnerable to financial scams
	11	Taken in by phone scams
	12	Taken in by a computer scam
nfvPPA	1	Gave bank details to scammer
	2	Taken in by scam emails about 'brain food'
AD	1	Caught out by online money scams
	2	Caught out by email scam
	3	Victim of an email scam

The table shows representative free text comments made by patients' primary caregivers as examples of inappropriate trusting behaviour (increased gullibility) since onset of the illness. AD, typical Alzheimer´s disease; bvFTD, behavioural variant frontotemporal dementia; nfvPPA, non-fluent primary progressive aphasia; svPPA, semantic variant primary progressive aphasia.

Table S2. Predictors of inappropriate trusting behaviour in patient groups

**Parameter**	**AD**	**bvFTD**	**svPPA**
No. of cases	27	25	17
MMSE	**0.71 (0.29-0.92)**	0.93 (0.67-1.24)	0.96 (0.42-1.31)
WASI Matrices score	0.94 (0.61-1.31)	0.99 (0.79-1.2)	0.99 (0.37-1.34)
RSMS-total	0.89 (0.5-1.0)	1.0 (0.87-1.12)	0.87 (0.45-1.03)
RSMS-EX	0.88 (0.41-1.17)	0.99 (0.76-1.17)	0.77 (0.38-1.06)
RSMS-SP	**0.71 (0.39-0.88)**	1.03 (0.8-1.42)	0.78 (0.42-1.17)
Obsessionality	1.10 (0.43-2.85)	0.92 (0.35-2.36)	1.0 (0.39-3.07)
Disinhibition	**0.60 (0.34-0.99)**	**2.24 (1.25-4.50)**	1.3 (0.52-3.52)
Apathy	0.65 (0.23-2.13)	1.75 (0.64-3.97)	**2.48 (1.03-5.14)**
Altered pain responsiveness	1.43 (0.68-3.34)	**3.83 (1.52-8.41)**	2.59 (0.97-5.58)

The table presents the odds ratios (with 95% confidence intervals) for general cognitive and socio-emotional behavioural symptoms as predictors of inappropriate trusting behaviour within different diagnostic groups. Significant odd ratios are in bold (all $p < 0.05$). The results here are based on logistic regression models in 69 patients with complete correlative neuropsychological and behavioural data (excluding the nfvPPA group, as only four patients with this diagnosis showed inappropriate trusting); this subcohort did not differ significantly in cognitive or behavioural characteristics from the full cohort assessed for the effect of diagnosis on financial vulnerability (see text and Supplementary Tables S1 and S3). AD, patient group with typical Alzheimer's disease. bvFTD, patient group with behavioural variant frontotemporal dementia; MMSE, Mini-Mental State Examination score; n, number of participants; RSMS-EX, Revised Self-Monitoring Scale - sensitivity to socio-emotional expressiveness subscore; RSMS-SP, ability to modify self-presentation subscore; svPPA, patient group with semantic variant primary progressive aphasia; WASI, Wechsler Abbreviated Scale of Intelligence.

Table S3. General demographic, neuropsychological and behavioural characteristics of the patient subcohort included in the predictor analysis

Characteristic	AD	bvFTD	svPPA
**Demographics**			
No. (m:f)	27 (12:15)	25 (18:7)	17 (10:7)
Handedness (R:L)	24:3	24:1	17:0
Age (y)	70.1 (7.5)	65.7 (7.0)	67.0 (7.3)
Education (y)	16.0 (12.5-16.0)	14.0 (12.0-16.0)	16.0 (11.2-16.0)
Symptoms duration (y)	6.1 (4.4-7.6)	4.9 (3.7-5.9)	5.7 (2.2)
MMSE (/30)	18.0 (16.5 -24.5)	24.0 (21.0-27.0)	23.5 (18.5-28.5)
WASI VIQ	92.0 (83.5-109.5)	82.0 (60.5-107.0)[a]	66.0 (55.0-76.0)
WASI PIQ	80.0 (71.0-93.5)	92.0 (82.0-106.0)[a]	108.0 (98.0-129.0)
**Neuropsychology**			
Episodic memory			
RMT Faces (/50)	27.0 (25.5-32.5)[h]	31.0 (25.0-39.0)	28.5 (27.3-35.5)[a]
RMT Words (/50)	28.0 (24.5-31.5)[h]	34.0 (27.5-44.0)[b]	32.0 (26.0-39.0)[b]
Executive			
DS forward (12)	6.0 (5.0-7.0)	6.0 (5.0-7.0)	7.0 (6.0-7.0)
DS reverse (12)	3.5 (3.0-4.0)[a]	4.0 (3.0-5.0)	5.0 (4.0-5.0)
WASI Matrices (30)	10.0 (6.5-15.5)	16.0 (9.0-21.0)	24.0 (21.0-28.0)
DKEFS Stroop: colour (90 s)	54.5 (45.0-62.0)[c]	46.5 (34.5-66.3)[a]	42.0 (36.0-65.0)
words (90 s)	33.0 (29.8-38.8)[c]	30.0 (22.8-35.3)[a]	28.0 (21.0-35.0)
interference (180s)	180.0 (114.5-180.0)[c]	87.0 (63.8-180.0)[a]	80.0 (59.0-121.0)
TMT-A (s)	86.0 (61.3-144.3)[a]	56.0 (42.3-96.0)[a]	53.0 (33.0-61.0)
TMT-B (s)	300.0 (285.5-300.0)[a]	196.0 (103.8-300.0)[a]	115.0 (82.0-177.0)
Letter fluency (F)	9.5 (6.0-11.8)[a]	8.0 (2.0-12.5)[a]	7.0 (5.0-12.0)
Category fluency (animals)	7.0 (5.0-11.5)	9.0 (4.8-16.3)[a]	6.0 (3.0-10.0)
Language			
BPVS (/150)	144.0 (124.0-146.5)	140.5 (129.3-148.0)[a]	91 (44.0-107.0)
GNT (/30)	14.5 (5.5-19.8)[a]	15.0 (3.0-24.0)	0 (0-0)
Other skills			
GDA (/24)	2.0 (0.3-4.0)[a]	6.0 (4.0-11.8)[c]	12.0 (5.0-15.0)
VOSP (/20)	16.0 (14.0-17.5)	15.0 (10.0-18.0)[b]	16.0 (15.0-17.0)
Social cognition			
RSMS-total	34.2 (12.5)	20.4 (11.8)	23.6 (11.5)
RSMS-EX	15.0 (10.0-21.0)	8.0 (2.0-14.0)	8.0 (5.0-12.0)
RSMS-SP	19.1 (6.6)	12.1 (5.4)	14.5 (6.1)
Obsessionality	7 (26)	20 (80)	12 (71)
Disinhibition	5 (19)	22 (88)	13 (76)
Apathy	18 (67)	20 (80)	8 (47)
Altered pain sense	3 (11)	12 (48)	11 (64)
Inappropriate trust	6 (22)	10 (40)	11 (64)

The table presents data for the patient subcohort with complete neuropsychological and general behavioural data, included in the analysis to identify predictors of inappropriate trusting behaviour (see main text). The nfvPPA group has been omitted here due to the small number of patients in this group exhibiting inappropriate trusting behaviour. This subcohort did not differ significantly on any cognitive or behavioural characteristic from the full patient cohort described in Table 1 (all

comparisons $p > 0.05$). Counts (standard deviation) are shown for general demographic and clinical data; mean (standard deviation) or median (interquartile range) scores are shown for neuropsychological tests (also with maximum scores in parentheses); and raw counts (percentage of group) are shown for behavioural change data. AD, patient group with typical Alzheimer's disease; BPVS, British Picture Vocabulary Scale (Dunn, Dunn and Whetton, 1982); bvFTD, patient group with behavioural variant frontotemporal dementia; D-KEFS, Delis Kaplan Executive System (Delis et al., 2001); DS, Digit Span; EX, sensitivity to socio-emotional expressiveness; f, female; GDA, Graded Difficulty Arithmetic test (Jackson and Warrington, 1986); GNT, Graded Naming Test (McKenna and Warrington, 1983); L, left; m, male; MMSE, Mini-Mental State Examination score (Folstein, Folstein and McHugh, 1975); PIQ, performance IQ; R, right; RMT, Recognition Memory Test (Warrington, 1984); RSMS, Revised Self-Monitoring Scale; s, seconds; SP, ability to modify self-presentation; svPPA, patient group with semantic variant primary progressive aphasia; Symptoms, estimated duration of symptoms since onset; TMT, trail making test; VIQ, verbal IQ; VOSP, Visual Object and Space Perception Battery – Object Decision test (Warrington, McKenna and Orpwood, 1998); WASI, Wechsler Abbreviated Scale of Intelligence (Wechsler, 1997). A reduced number of patients completed certain tests, as follows: [a]n-1, [b]n-2, [c]n-3, [h]n-8.
